# Single Intratracheal Quartz Instillation Induced Chronic Inflammation and Tumourigenesis in Rat Lungs

**DOI:** 10.1038/s41598-020-63667-4

**Published:** 2020-04-20

**Authors:** Yuko Nakano-Narusawa, Masanao Yokohira, Keiko Yamakawa, Kousuke Saoo, Katsumi Imaida, Yoko Matsuda

**Affiliations:** 1 Oncology Pathology, Department of Pathology and Host-Defence, Faculty of Medicine, Kagawa University, Kagawa, 761-0793 Japan; 2 Kaisei General Hospital, Kagawa, 762-0007 Japan

**Keywords:** Chemical biology, Cancer, Lung cancer

## Abstract

Crystalline silica (quartz) is known to induce silicosis and cancer in the lungs. In the present study, we investigated the relationship between quartz-induced chronic inflammation and lung carcinogenesis in rat lungs after a single exposure to quartz. F344 rats were treated with a single intratracheal instillation (i.t.) of quartz (4 mg/rat), and control rats were treated with a single i.t. of saline. After 52 or 96 weeks, the animals were sacrificed, and the lungs and other organs were used for analyses. Quartz particles were observed in the lungs of all quartz-treated rats. According to our scoring system, the lungs of rats treated with quartz had higher scores for infiltration of lymphocytes, macrophages and neutrophils, oedema, fibrosis, and granuloma than the lungs of control rats. After 96 weeks, the quartz-treated rats had higher incidences of adenoma (85.7%) and adenocarcinoma (81.0%) than control rats (20% and 20%, respectively). Quartz-treated and control rats did not show lung neoplastic lesions at 52 weeks after treatment. The number of lung neoplastic lesions per rat positively correlated with the degree of macrophage and lymphocyte infiltration, oedema, fibrosis, and lymph follicle formation around the bronchioles. In conclusion, single i.t. of quartz may induce lung cancer in rat along with chronic inflammation.

## Introduction

Silica (silicon dioxide, SiO_2_, or quartz) takes either a crystalline or an amorphous form depending on the environmental pressure and temperature. Amorphous silica is classified as a Group 3 agent, or an agent that has inadequate evidence of carcinogenicity, by the International Agency for Research on Cancer (IARC)^1^; therefore, it is widely used in food and cosmetics. Crystalline silica (quartz, CAS No. 14808-60-7), on the other hand, is classified as a Group 1 agent or a human carcinogen^1,2^. Quartz is commonly distributed in mineral. Exposure to quartz in atmospheric dust occurs in nature, industrial activity, and farming.

Silicosis is induced by chronic occupational exposure to quartz^3–5^ and found in individuals such as mining^2,6^, ceramic^7^, and artificial quartz conglomerate workers^8^, who are involved in the manufacture, finishing, and installation of artificial stone countertops^9^. The risk of silicosis induced by occupational exposure to 0.05–0.10 mg/m^3^ of quartz in a lifetime has shown large variability (2–90%). Silicosis is a non-neoplastic reaction to the deposition of quartz in the lungs and is classified into the following phases: acute (develops within weeks to a few years), accelerated (develops within 10 years), and chronic (develops more than 10 years after initial exposure)^10^. The lesions are characterised by micronodular scars along the lymphatic network, particularly around bronchovascular bundles in humans. Early lesions appear as cellular nodules composed of fibroblasts and histiocytes that contain large amounts of silica particles; older nodules are hyalinised and less cellular^3^. An autopsy study showed that silicosis is associated with high frequencies of adenocarcinoma and squamous cell carcinoma but not of small cell carcinoma^10^.

A significant positive relationship between cumulative silica exposure and lung cancer mortality has already been reported^11^; in this study, 1,079 of 65,980 silica-exposed workers died of lung cancer. The risk of lung cancer increases significantly when an individual’s cumulative exposure to silica exceeds 1.8 mg/m^3^, with the risk nearly doubling among workers exposed to >6.0 mg/m^3^ of quartz^12^. Other than cumulative exposure, various factors have been reported to be related to lung cancer risk, including the duration of exposure^13^, the degree of peak exposure^14^, silicosis determined by X-ray^15^, and the period from diagnosis of silicosis^16^. Although previous studies did not distinguish the effects of cigarette smoke, the relative risks of lung cancer in people exposed to quartz were similar regardless of smoking history^2,17,18^. The toxicity and carcinogenicity of quartz depend on the characteristics of the quartz particles, such as size, surface properties, and area of production. The IARC Working Group noted that carcinogenicity to humans was not detected in any of the industrial circumstances studied and that carcinogenicity may be dependent on inherent characteristics of the crystalline silica or on external factors affecting its biological activity^19^.

Inflammatory changes induced by quartz exposure depend on the animal species. DQ12 (quartz sand consisting of 87% quartz and 13% amorphous silica kaolinite)^20^, Min-U-Sil (99% quartz), and novaculite (a variety of microcrystalline quartz)^17^ have been commonly used in animal studies. We have reported that the lungs of A/J mice that were subjected to intratracheal instillation (i.t.) of quartz (DQ12) had milder inflammatory changes than the lungs of quartz-treated F344 rats^21^. Comparative histopathological studies of rats and humans exposed to quartz revealed that rats had more severe inflammation and alveolar epithelial hyperplasia than humans^22,23^. Previous studies showed that quartz exposure induced lung tumourigenesis in rats but not in mice or hamsters^2,24^. We have reported that F344 rats showed very severe chronic inflammation and N-nitrosobis (2-hydroxypropyl)amine (DHPN)-induced lung carcinogenesis following quartz instillation, unlike Sprague-Dawley and Wistar-Hannover rats^25^. The F344 strain was sensitive to carcinogenesis induced by quartz and can be used to model the effects of quartz on the lungs^26–29^.

Quartz particles, which have a diameter smaller than 3–4 μm, can reach alveolar epithelial cells and stromal cells and then move to the lymph nodes. Impairment of quartz clearance by alveolar macrophages due to quartz-induced toxicity and/or inflammation enhanced the production of cytokines, chemokines, reactive oxygen, reactive nitrogen, and hydroxyl radicals by macrophages and neutrophils which, in turn, led to epithelial cell injury and proliferation^2,10,30–32^. However, it has not been reported whether quartz is mutagenic, and its genotoxicity, although not directly proven, has not been refuted in IARC reports. Type II alveolar cells may proliferate in response to chronic inflammation and form hyperplastic lesions^33^. We have reported that reactive hyperplastic-like changes and hyperplasia (pre-neoplastic/precursor lesions) in rodent lungs showed similar morphological characteristics^25,34,35^, but immunostaining of surfactant protein C (SP-C) and napsin A could be used to distinguish inflammatory changes and pre-neoplastic lesions^36,37^. Borm *et al*.^24^. reviewed the different modes of action of respirable crystalline silica-induced genotoxicity in a series of independent studies. In *in vitro* studies, the results of comet assay were mostly negative, apart from two studies that used primary or cultured macrophages. *In vivo* studies confirmed the role of persistent inflammation due to quartz surface toxicity, which led to anti-oxidant responses in mice and rats; nonetheless, DNA damage was only observed in rats.

There are many reports on the effects of multiple i.t. and few reports on single i.t. exposure. In particular, reports concerning the long-term toxicity of a single i.t. of quartz are lacking. In the present study, we investigated the relationship between quartz-induced chronic inflammation and carcinogenesis for approximately 2 years (almost the entire life span of rats) in F344 rats treated with a single i.t. of quartz without initiation by a carcinogen.

## Results

### Body and organs weights

Fifty-two 8-week-old male F344 rats were randomly assigned to four groups (Table 1). Groups 52w-quartz and 96w-quartz were given a single i.t. of 4 mg of quartz (DQ12) in a saline solution (0.2 ml), while groups 52w-saline and 96w-saline were given the vehicle control (saline). In our previous study^38,39^, we used DQ-12 (4 mg/0.2 ml saline per rat) to detect lung toxicity due to fine particles in F344 male rats; the lungs treated with this dose of DQ-12 exhibited severe inflammatory changes 28 days after i.t., and therefore this dose was used in the present study. The long-term effects of a single i.t. exposure to quartz on the body and organ (lung, kidney, liver, and spleen) weights were determined after 52 (groups 52w-quartz and 52w-saline) or 96 (groups 96w-quartz and 96w-saline) weeks. One rat from group 52w-quartz (at week 51), four rats from group 96w-quartz (at weeks 73, 81, 91, and 92), and four rats from group 96w-saline (at weeks 59, 85, 92, and 94) died before the end of the experimental period, and these animals were excluded from subsequent analyses. These nine rats did not appear grossly weakened before death. At week 95, 4 rats from group 96w-quartz were sacrificed. The 4 rats were considered moribund because of symptoms such as general weakness, hair loss, and bleeding from the mass of the skin. The remaining rats in groups 96w-quartz and 96w-saline were sacrificed at 96 weeks. The final evaluation included 15 rats (nine rats in group 52w-quartz and six rats in group 52w-saline) on week 52, four rats (group 96w-quartz) on week 95, and 22 rats (17 rats in group 96w-quartz and five rats in group 96w-saline) on week 96. The absolute and relative (body weight-normalised) lung weights and the relative kidney weights of the rats from group 52w-quartz were significantly higher than those of the rats from group 52w -saline (P < 0.05, Table 1). The absolute lung weights of group 96w-quartz rats were significantly higher than those of group 96w-saline rats (P < 0.01, Table 1). There were no significant differences in the body weights and in the absolute and relative weights of liver and spleen between the groups, but the rats of groups 52w-quartz and 96w-quartz had generally lower body weights than those of groups 52w-saline and 96w-saline. These results demonstrated that quartz exposure affects the integrity of rat lungs and kidneys long term.

**Table 1:** Body and organ weights and incidences of existence of quartz in the lungs.

Group	Treatment	Experimental period (weeks)	Age at sacrifice (weeks)	No. of rats	Body weight (g)	Lung		Liver		Kidney		Incidences of existence of quartz in the lung (%)
						Absolute (g)	Relative (%)	Absolute (g)	Relative (%)	Absolute (g)	Relative (%)	
52w-quartz	Quartz i.t.	52	60	9(10)	385.5 ± 14.8	1.8 ± 0.3*	0.5 ± 0.1*	10.2 ± 0.7	2.6 ± 0.1	2.2 ± 0.1	0.6 ± 0.0*	100** (9/9)
52w-saline	Saline i.t.	52	60	6(6)	398.0 ± 17.3	1.5 ± 0.2	0.4 ± 0.1	10.7 ± 0.4	2.7 ± 0.1	2.1 ± 0.1	0.5 ± 0.1	0 (6/6)
96w-quartz	Quartz i.t.	95 or 96	103 or 104	21(26)	303.5 ± 63.4	3.5 ± 0.6**	1.3 ± 0.5	8.3 ± 1.7	2.8 ± 0.4	2.1 ± 0.2	0.7 ± 0.2	100** (21/21)
96w-saline	Saline i.t.	96	104	5(10)	321 ± 76.0	2.6 ± 0.9	0.8 ± 0.3	9.1 ± 1.9	2.8 ± 0.1	2.2 ± 0.1	0.7 ± 0.2	0 (5/5)

^*^Denotes significant differences compared with the saline i.t. group (P < 0.05).

^**^Denotes significant differences compared with the saline i.t. group (P < 0.01).

The numbers between parentheses in the column ‘No. of rats’ indicate the original number of rats.

### Quartz particles in the lungs

**Figure 1:**
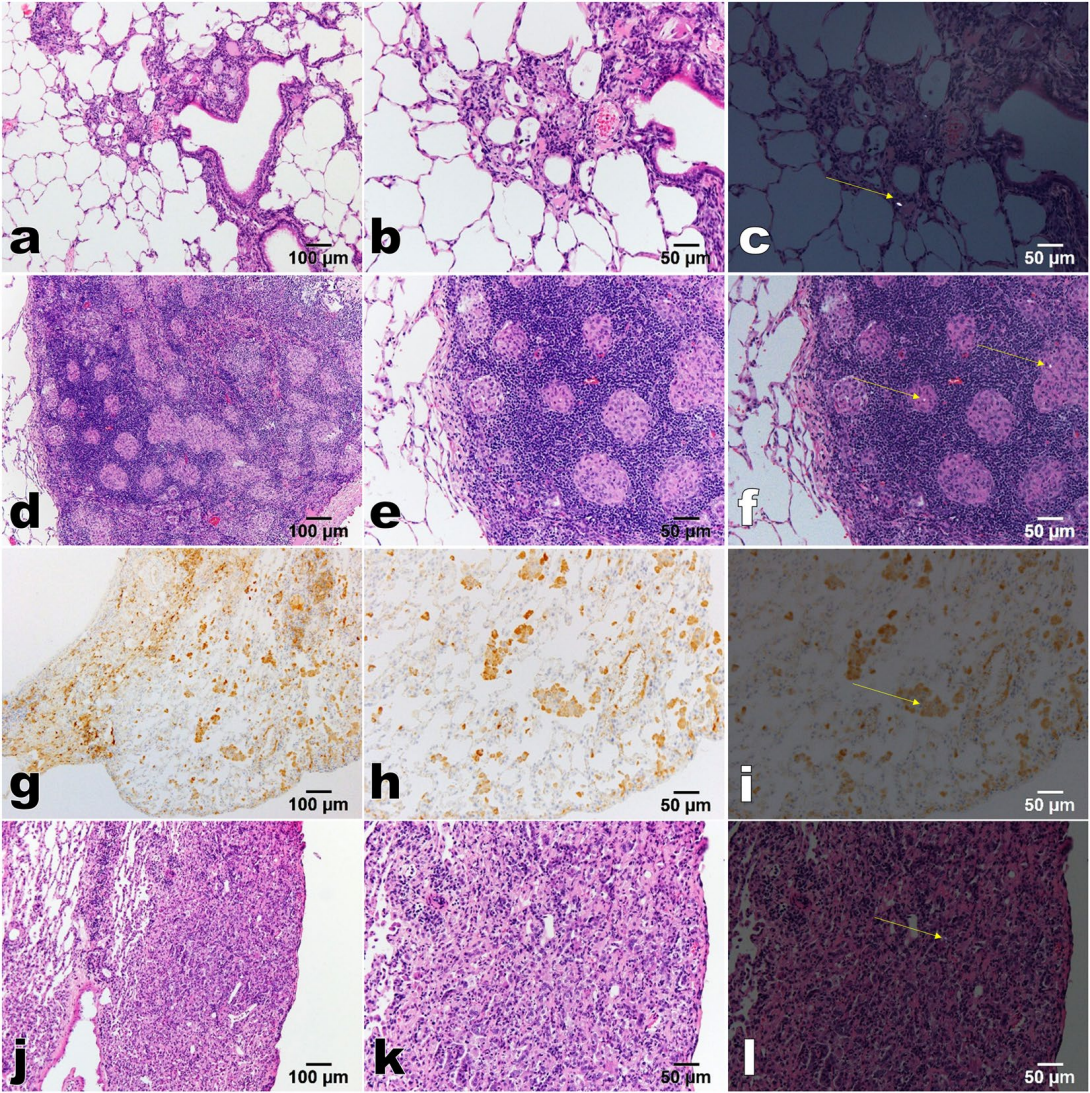
Quartz particles in the lungs of rats 95–96 weeks after i.t. (group 96w-quartz). (**a**–**f**,**j**–**l)**, **H**&**E** staining; **(g-i)**, immunohistochemical staining of CD68; (**a,d,g,j)**, low magnification (100×); **(b**,**c,e,f,h,i,k**,**l**), high magnification (200×); (**c,f,i,l**), polarisation microscopy (yellow arrow, quartz particle). (**a-c)**, In the fibrosis area; (**d**–**f)**, in the granulation tissue; (**g**–**i)**, in a macrophage; (**j**–**l**), in the adenoma.

Quartz particles in the lungs of groups 52w-quartz and 96w-quartz were observed by polarised light microscopy (P = 0.0001, Table 1). Collagen was also visible under the polarised microscope, but it could be distinguished from quartz particles based on morphological differences: collagen appeared thin and with band-like fibres, and quartz particles were spherical. The incidence of quartz particles in the lungs refers to the number of rats with the confirmed presence of quartz particles in the lungs among the total number of rats. Quartz particles were observed in the fibrotic area (Fig. 1a-c), in the granuloma (Fig. 1d-f), in macrophages in the alveolar space (Fig. 1g,h,i, positive for CD68), and in neoplastic lesions (Fig. 1j-l, adenoma of the lung). These results suggested that quartz particles remain in the lung 52 or 96 weeks after a single i.t.

### Inflammatory changes

**Figure 2:**
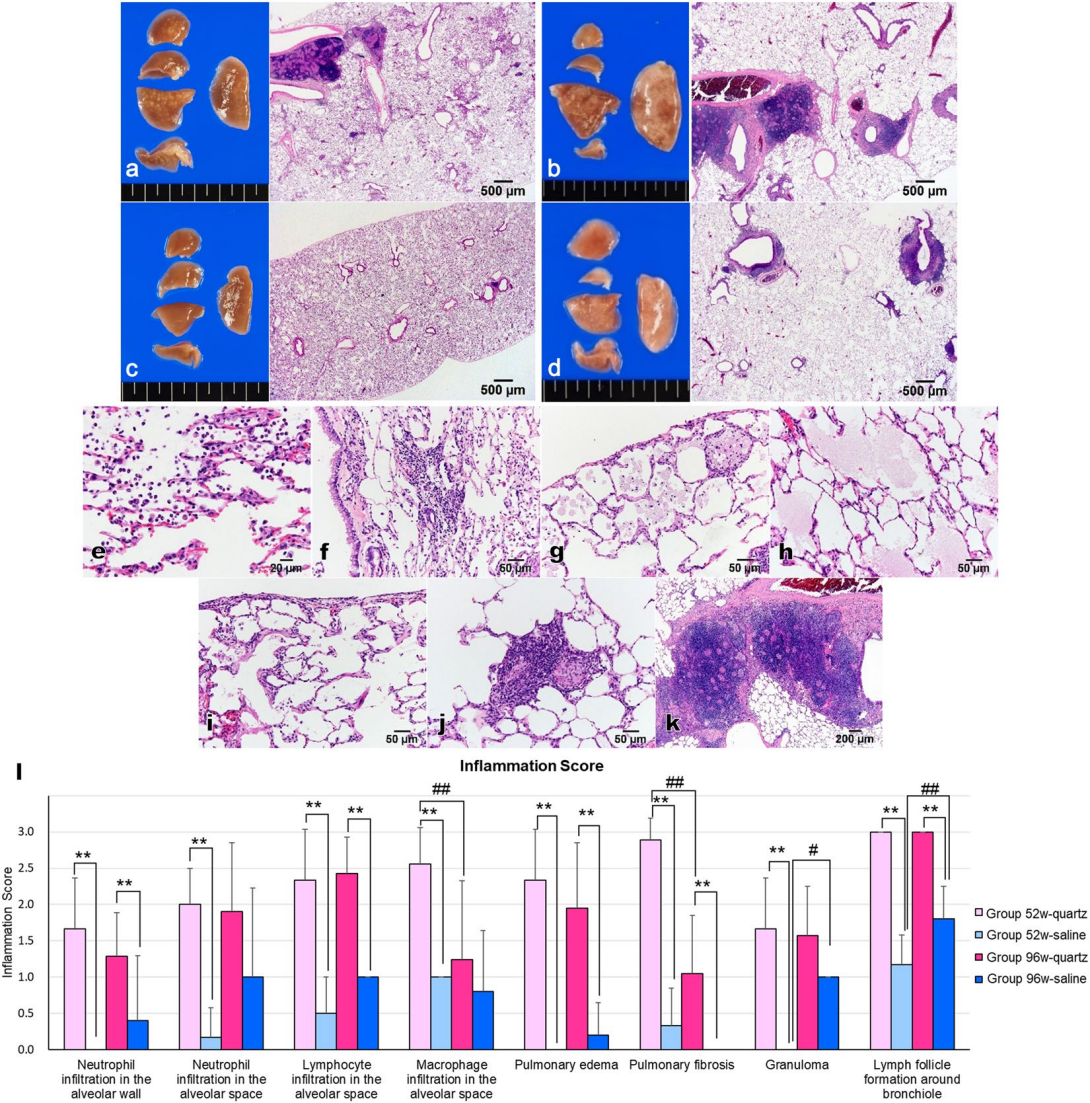
Macroscopic findings (**a-d**) and histopathological inflammatory findings (**e-k**) and scores (**l**). (**a**) Group 52w-quartz; (**b**), group 96w-quartz; (**c**), group 52w-saline; (**d**), group 96w-saline; (**e**), neutrophil infiltration in the alveolar walls and in the alveolar space; (**f**), lymphocyte infiltration in the alveolar space; (**g**), macrophage infiltration in the alveolar space; (**h**), pulmonary oedema; (**i**), pulmonary fibrosis; (**j**), granuloma; (**k**), lymph follicle formation around the bronchiole; (**l**), inflammation score. **Significant differences compared to the saline i.t. group (P < 0.01). ^##^Significant differences compared to the 52w-quartz i.t. group (P < 0.01). Data presented are means ± SD.

Whitish nodules were observed macroscopically in the lungs of rats from groups 52w-quartz and 96w-quartz (Fig. 2a,b), while there were no significant findings in the lungs of rats from groups 52w-saline and 96w-saline (Fig. 2c,d). Histopathologically, the lungs of rats from groups 52w-quartz and 96w-quartz showed severe inflammatory changes in the alveolar area and lymph follicle formation around the bronchiole (Fig. 2a,b), while the lungs of rats from groups 52w-saline and 96w-saline had only modest inflammatory changes (Fig. 2c,d). The inflammatory changes in groups 52w-quartz and 96w-quartz were observed in the central and peripheral areas of all lobes of the lungs.

The lungs from groups 52w-quartz and 96w-quartz showed neutrophil infiltration in the alveolar walls and in the alveolar space (Fig. 2e), lymphocyte infiltration (Fig. 2f), macrophage infiltration (Fig. 2g), pulmonary oedema (Fig. 2h), pulmonary fibrosis (Fig. 2i), granuloma (Fig. 2j), and lymph follicle formation around the bronchioles (Fig. 2k). These changes were absent or weak in the lungs from groups 52w-saline and 96w-saline. We evaluated the inflammation score (Fig. 2l and Supplementary Table 1) as previously reported^25^. Briefly, inflammatory changes in the lungs were scored using haematoxylin and eosin (H&E)-stained specimens based on neutrophil infiltration in the alveolar wall or alveolar space; lymphocyte infiltration in the alveolar space; macrophage infiltration in the alveolar space; pulmonary oedema; pulmonary fibrosis; granuloma; and lymph follicle formation around the bronchioles. The rats from group 52w-quartz had significantly higher inflammation scores than the rats from group 52w-saline (P < 0.01, Fig. 2l). Group 96w-quartz rats also had significantly higher inflammation scores than group 96w-saline rats in most evaluations (P < 0.01, Fig. 2l); however, there were no statistical differences in the scores for macrophage and neutrophil infiltration in the alveolar space and for granuloma between groups 96w-quartzand 96w-saline. Macrophage infiltration and pulmonary fibrosis in group 96w-quartzwere significantly lower than those in group 52w-quartz (P < 0.01, Fig. 2l). Granulomas and lymph follicle formation around the bronchiole in group 96w-saline were significantly more prevalent than those in group 52w-saline (P < 0.05, P < 0.01, Fig. 2l). These results suggested that quartz-induced inflammation remains in the lungs 52 or 96 weeks after a single i.t.

### Hyperplastic and neoplastic lesions in the lungs

**Figure 3:**
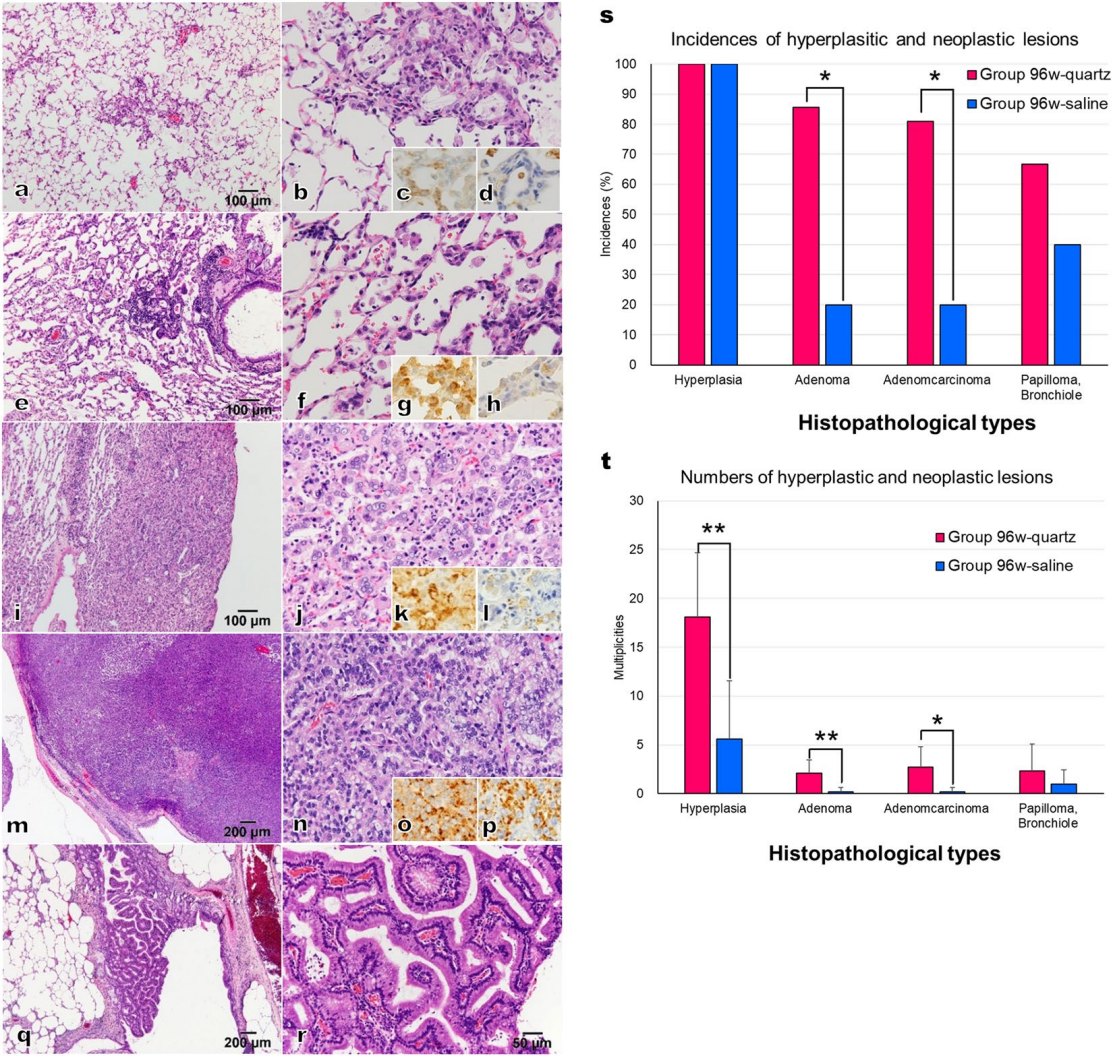
Hyperplastic and neoplastic lesions in quartz-treated groups. (**a-d**) Inflammatory change (group 52w-quartz); (**e-h**), hyperplasia (group 96w-quartz); (**i-l**), adenoma (group 96w-quartz); (**m-p**) adenocarcinoma (group 96w-quartz); (**q,r**), papilloma, bronchiole (group 96w-quartz); **(a**,**e**,**i**,**m**,**q**), low magnification; (**b,f,j,n,r**) high magnification; (**c,d,g,h,k,l,o,p**), immunohistochemical assessments; (**c,g,k,o**), SP-C; (**d,h,l,p**), napsinA; (**s)**, incidences of hyperplastic and neoplastic lesions; (**t**), numbers of hyperplastic and neoplastic lesions per rat. *, **Significant differences compared with the saline i.t. group (P < 0.05, <0.01, respectively). Data presented are means ± SD.

Groups 52w-quartz and 52w-saline exhibited inflammatory changes (Fig. 3a-d) but did not display hyperplastic or neoplastic lesions in the lungs (Supplementary Table 2). Groups 96w-quartz and 96w-saline showed several lesions of hyperplasia (Fig. 3e-h), adenoma (Fig. 3i-l), adenocarcinoma (Fig. 3m-p), and papilloma (Fig. 3q,r). Similar to previous reports^35–37^, inflammatory changes were negative for napsin A, whereas hyperplasia, adenoma, and adenocarcinoma were positive for napsin A. The incidences of adenoma and adenocarcinoma in the lungs from group 96w-quartz were significantly higher than those in the lungs from group 96w-saline (P < 0.05; Fig. 3s, Supplementary Table 3). The numbers of hyperplasia, adenoma, and adenocarcinoma per rat in group 96w-quartz were significantly higher than those in group 96w-saline (P < 0.05, P < 0.01; Fig. 3t, Supplemental Data 3). Papillomas in the bronchioles were observed in both 96w-quartzand 96w-saline rats, and there were no significant differences in the incidence and the number of lesions per rat between the two groups (Fig. 3t).

**Figure 4:**
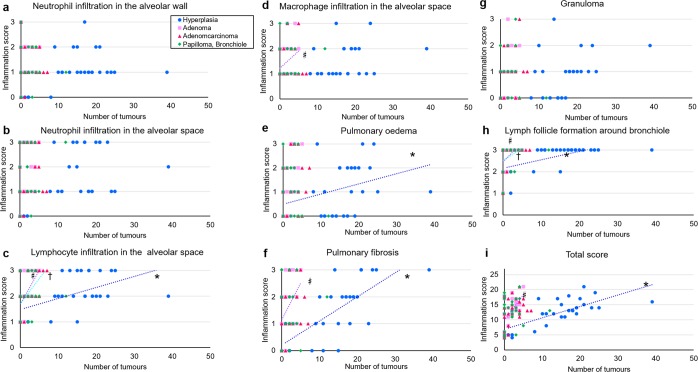
The correlations between inflammation score and the numbers of hyperplastic and neoplastic lesions per rat at 96 weeks. One point corresponds to the score of one rat. (**a**) Neutrophil infiltration in the alveolar wall; (**b**), neutrophil infiltration in the alveolar space; (**c**), lymphocyte infiltration in the alveolar space; (**d**), macrophage infiltration in the alveolar space; (**e**), pulmonary oedema; (**f**), pulmonary fibrosis; (**g**), granuloma; (**h**), lymph follicle formation around the bronchiole; (**i**), total score. R = Spearman’s correlation coefficient, P = P value. *Significant correlations with number of hyperplasia. ^#^Significant correlations with number of adenomas. ^†^Significant correlations with number of adenocarcinomas. The following show significant differences in each combination. c*: R = 0.47, P = 0.01; **c**^#^: R = 0.53, P = 0.01; **c**^†^: R = 0.56, P = 0.002; **d**^#^: R = 0.40, P = 0.05; e*: R = 0.69, P = 0.0001; f*: R = 0.50, P = 0.01; **f**^#^: R = 0.46, P = 0.02; h*: R = 0.61, P = 0.001; **h**^#^: R = 0.50, P = 0.01; **h**^†^: R = 0.45, P = 0.02; i*: R = 0.69, P = 0.0001; **i**^#^: R = 0.57, P = 0.002.

At 96 weeks, some parameters of the inflammation score were positively correlated with the number of lesions per rat of hyperplastic and neoplastic lesions (Fig. 4). Macrophage infiltration in the alveolar space and oedema were positively correlated with the numbers of lesions per rat, hyperplasia (R = 0.69, P = 0.0001) and adenoma (R = 0.40, P = 0.05), respectively. Lymphocyte infiltration in the alveolar space and lymph follicle formation around the bronchiole were positively correlated with the numbers of lesions per rat, hyperplasia (R = 0.47, P = 0.01; R = 0.61, P = 0.001), adenoma (R = 0.53, P = 0.01; R = 0.50, P = 0.01), and adenocarcinoma (R = 0.56, P = 0.003; R = 0.45, P = 0.02). Pulmonary fibrosis and the total inflammation score were positively correlated with the numbers of lesions per rat, hyperplasia (R = 0.50, P = 0.01; R = 0.69, P = 0.0001) and adenoma (R = 0.46, P = 0.02; R = 0.57, P = 0.002). These results suggested that lung inflammation due to quartz exposure is positively associated with lung carcinogenesis.

### Other organ changes

Several lesions in organs other than the lungs were detected in group 96w-quartz but not in the other groups. Neoplastic changes in this group included cholangiocarcinoma (n = 1), fibroma of the skin (n = 3), sebaceous adenoma of the skin (n = 1), adenocarcinoma of the mammary gland (n = 1), Leydig cell tumour of the testis (n = 1), adenoma of the testis (n = 1), and benign pheochromocytoma of the adrenal glands (n = 5). Non-neoplastic changes included fatty change and granulomatous change in the liver; lymphocyte infiltration, pyelonephritis, tubular necrosis, hypertrophy and congestion in the kidney; and necrosis of the spleen (Supplemental Data 4). Quartz particles were not detected in these organs.

Groups 52w-quartz and 52w-saline exhibited bile duct hyperplasia and lymphocyte infiltration, clear cell foci in the liver, and tubule regeneration and lymphocyte infiltration in the kidney. Groups 96w-quartz and 96w-saline had bile duct hyperplasia, lymphocyte infiltration, fibrosis, basophilic foci, and clear cell foci in the liver; tubular regeneration, casts, chronic progressive nephropathy, pigmentation of hemosiderin, and cysts in the kidney; and congestion and pigmentation of hemosiderin in the spleen. Clear cell foci in the liver and chronic progressive nephropathy in the kidney in group 96w-quartz were significantly lower incidences than those found in group 96w-saline. There were no significant differences in the incidences of other lesions between groups. According to these results, the effects on organs other than the lung were not apparent with a single i.t.

## Discussion

The present study revealed the following findings: (1) quartz particles remained in the lungs for 96 weeks after only a single i.t. in male F344 rats, (2) quartz induced persistent chronic inflammation of the lungs for 96 weeks, and (3) quartz induced carcinogenesis of the lungs in 85.7% of the rats by 96 weeks but not 52 weeks after instillation. Our study further elucidated the persistence and carcinogenicity of quartz particles in the lungs.

Previous studies with F344 rats have shown that a single i.t. dose of 20 mg of quartz (Min-U-Sil or novaculite) induced lung tumours in 44.8% of Min-U-Sil-instilled male rats and in 29.2% of novaculite-treated male rats 96 weeks after instillation^29^. Moreover, a single 12-mg Min-U-Sil instillation induced lung tumours in 54.5% of the male rats and in 69.2% of the female rats by 74 weeks, and a single 20-mg instillation induced lung tumours in 75% of the female rats^40^. Of note, these doses of quartz are higher than those used in our present study. Albrecht *et al*. reported that, after a single i.t. of 2 mg of DQ12(low dose) in female Wistar rats, 33% of the instilled quartz remained in the lungs 90 days after instillation. Particle surface-specific interactions between the quartz and macrophages were determined to play a major role in the pulmonary pathogenicity of the quartz. However, the study evaluated short-term reactivity and did not assess tumour formation^41^. In the present study, the experimental period lasted 2 years, and the dose of quartz was 4 mg/rat, which we expected to cause a persistent inflammatory response and enable evaluation of possible tumour formation. This dose induced severe inflammatory changes 28 days after i.t. in our previous study^25^, and sufficient amounts of quartz were expected to remain in the lungs 2 years after exposure. Quartz particles were indeed observed in macrophages and granulation or fibrotic tissues in the lungs 96 weeks after quartz i.t. Chronic inflammation was observed in these tissues, which is considered to affect cell proliferation. Collectively, the previous reports and the present study indicate that the incidence of lung tumours depends on time and the dose and type of quartz particles.

The numbers of hyperplastic and neoplastic lesions per rat were positively correlated with macrophage and lymphocyte infiltration in the alveolar space and lymph follicle formation around the bronchioles. In addition, quartz-treated rats had higher scores for neutrophil infiltration than the control rats. Freire *et al*. reported that quartz-mediated chronic inflammation created a microenvironment that favoured tumour development in mice treated with the carcinogen N-nitrosodimethylamine (NDMA) and quartz through the involvement of regulatory T-cells and the escape of cancer cells from immune elimination^42^. In the present study, the scores for macrophage infiltration and pulmonary fibrosis were higher in rats observed 52 weeks after quartz i.t. than in rats observed 96 weeks after quartz treatment, suggesting that clearance of quartz and repair of lung tissues were time-dependent. The total inflammation score was also decreased, suggesting that inflammation may have subsided.

The incidences of spontaneous lung proliferative lesions in 104-week-old male F344 rats have been previously reported as follows: adenoma (3%), adenocarcinoma (1%), squamous cell carcinoma (0.04%), and adenosquamous carcinoma (0.04%)^43^. In the present study, control rats had lower incidences of adenoma (20%) and adenocarcinoma (20%) than quartz-treated rats (86% and 81%, respectively) 96 weeks after i.t. The control and quartz-treated rats did not exhibit lesions 52 weeks after treatment, suggesting the influence of ageing in tumour progression. These incidences of adenoma and adenocarcinoma were higher than those described in the previous report. This may be because the number of animals in one of the groups was only five, and proliferative lesions were observed in one rat. In contrast, the incidences reported in the previous review are based on data from more than 100 rats. Chronic pro-inflammatory status is also a reported pervasive feature of ageing^44^. Consistent with this, the lungs of the control rats observed at 96 weeks after vehicle treatment had higher scores for granuloma and lymph follicle formation than those of the control rats observed at 52 weeks after vehicle treatment. Although the rats were not housed in specific pathogen-free (SPF) facilities, routine monitoring of microorganisms was performed, and microorganisms were not detected. Inflammation in the control groups might be related to ageing.

Livers and kidneys also showed inflammatory changes 96 weeks after treatment in the control and quartz-treated rats. Similar to previous reports^45,46^, we found spontaneous tumour growth in these organs; thus, we considered these observations unrelated to quartz exposure. There were age-related or spontaneous pathological changes in various organs, but chronic inflammation in the lungs due to quartz exposure persisted and overlapped with lung carcinogenesis.

The current study has several limitations. Quartz induced chronic inflammation and adenocarcinoma in rat lungs but not scarring, which is characteristic of silicosis in humans. Furthermore, we could not distinguish age-dependent changes and quartz-induced changes. However, our study clearly shows that there is an association between chronic inflammation and carcinogenesis. Inhibition of quartz-induced inflammation might be an effective approach to prevent cancer.

In conclusion, a single i.t. of quartz has carcinogenic potential in rat lungs with chronic inflammation. The results of this study may translate to human health and indicate that even a single exposure to quartz may have long-term effects on lung health. Precautions should therefore be taken, especially for people who are at risk of quartz exposure. Further studies on mitigating the effects of quartz exposure on the lungs are warranted.

## Methods

### Chemicals

Crystalline quartz particles (DQ12)^21,25,38,39^ were obtained from Deutsche MontanTechnologie, GmbH (Essen, Germany). The DQ12 particles in the present study have the following size properties: 1.707 μm (d50); 4.240 μm (d90); 0.252 μm (minimum diameter); 15.887 μm (maximum diameter). The particle size was analysed with LMS-2000e (SEISHIN ENTERPRISE Co., Ltd., Tokyo, Japan). Physiological saline (Otsuka isotonic sodium chloride solution from Otsuka Pharmaceutical Factory, Inc., Tokushima, Japan) was used as the vehicle for all test substances.

### Animals

Six-week-old male F344/DuCrlCrlj rats were purchased from Japan Charles River Inc. (Kanagawa, Japan). The rats were maintained in the Division of Animal Experiments, Life Science Research Center, Kagawa University, according to the Institutional Regulations for Animal Experiments, housed in wire cages, and given free access to drinking water and a basal diet of CE-2 (CLEA Japan Inc., Tokyo, Japan) under controlled conditions (humidity: 60 ± 10%; lighting: 12-hour light/dark cycles; and temperature: 24 ± 2 °C). The experiments were started after 2 weeks of acclimatisation^21,25,38,39,47^.

### Experimental design

A total of 52 male F344 rats (8 weeks old) were randomly divided into four groups of 6 to 26 rats per group (group 52 w-quartz: 10 rats; group 52w-saline: 6 rats; group 96w-quartz: 26 rats; group 96w-saline: 10 rats; Table 1) to minimise the number of animals. At the beginning of the experiment, the rats received a single i.t. of the following test solutions under anaesthesia: for groups 52w-quartz and 96w-quartz, each rat was given 4 mg quartz suspended in 0.2 ml saline; for the control groups 52w-saline and 96w-saline, each rat was given the vehicle solution (0.2 ml saline). In our previous study^38,39^, we used DQ-12 (4 mg/0.2 ml saline per rat) to detect lung toxicity due to fine particles in F344 male rats; the lungs treated with this dose of DQ-12 exhibited severe inflammatory changes 28 days after i.t., and therefore this dose was adapted in the present study.

I.t. was performed as follows. After anaesthetisation, each rat was placed on a table and fixed with three elastic bands hooked under the anterior teeth. The i.t. procedure was performed using a specially designed aerosoliser (PennCentury, Philadelphia, PA, USA) after wiping the intraoral mucosa with a cotton swab. The top of the insufflator was appropriately shaped to allow spray application of particles^25,38^. The i.t. procedure can spread various particles in the lungs by diffusion, similar to inhalation of chemicals in humans. The processing time was approximately 10 seconds; therefore, the animals did not suffer any injury, and there was no death related to the procedure in the present study.

Rats were sacrificed under anaesthesia after 52 weeks (groups 52w-quartz and 52w-saline) or 95 and 96 weeks (groups 96w-quartz and 96w-saline). The experimental design was approved by the Animal Care and Utilization Committee of Kagawa University, Japan (approval number #142). The animals were cared for in accordance with the institutional guidelines as well as the Guidelines for Proper Conduct of Animal Experiments.

### Tissue preparation

The lungs, trachea, heart, liver, and kidneys were removed during autopsy. At the sacrifice in the second year, the spleen, skin, mammary glands, adrenal glands, and testes were also removed from groups 92w-quartz and 92w-saline because macroscopic changes were observed in these organs. The lungs, including the trachea and heart, were weighed and infused with 10% phosphate-buffered formalin. After dissection, the remaining tracheas and hearts were weighed. The weights of the lungs were calculated by subtraction of these weights from the initial weight. The lungs were immersed in 10% phosphate-buffered formalin for one week, and two slices of the left lobe and one slice of the other lobe were embedded in paraffin for histopathological examination by H&E staining and immunohistochemical staining^21,25,38,39,47^. These slices were cut perpendicularly to the bronchi insofar as possible. Specimens from other organs were also processed for histopathological analyses.

### Immunohistochemical staining

The lungs of the rats were immunostained by the labelled streptavidin-biotin method using the Ventana Discovery staining system (Ventana Medical Systems, Tucson, AZ, USA) for the staining of SP-C, and the Leica BOND-III staining system (Leica Biosystems, Nussloch, Germany) for the staining of napsin A and CD68. The anti-rat SP-C polyclonal antibody (1:50 dilution; sc-13979, Santa Cruz Biotechnology, Inc., Santa Cruz, CA, USA); anti-rat napsin A monoclonal antibody (1:100 dilution; NCL-N-Napsin A, Leica Biosystems Newcastle Ltd., Newcastle Upon Tyne, UK); and anti-rat CD68 monoclonal antibody (1:100 dilution; M 0876, DakoCytomation, Glostrup, Denmark) were used. In our previous study^35–37^, the expression of napsin A in the alveolar walls was used to identify inflammatory changes, such as hyperplasia and lesions (negative for napsin A), which were thought to progress to neoplastic lesions (positive for napsin A; hyperplasias, adenomas, and adenocarcinomas). SP-C localises in type II alveolar cells^48^. In our previous study, we reported that high expression of SP-C is a potential marker of proliferative lesions including hyperplasia^35^. CD68 was used as a marker for macrophages because it is highly expressed by monocytes.

### Histopathological analysis

The lung lesions were categorised as hyperplasia (bronchiolo-alveolar), adenoma (bronchiolo-alveolar), papilloma (bronchiole), and adenocarcinoma (bronchiolo-alveolar) in accordance with the International Harmonization of Nomenclature and Diagnostic Criteria (INHAND)^49^. The incidences and the number of lesions per rat of each histopathological type are shown in Supplementary Table 2.

Previously, we published a scoring system for lung inflammatory changes in rats to enable statistical comparison of the inflammatory changes induced by i.t. of particles in different groups^25,38,47^. Inflammatory changes in the lungs were histopathologically examined for neutrophil infiltration in the alveolar wall or alveolar space; lymphocyte infiltration in the alveolar space; macrophage infiltration in the alveolar space; pulmonary oedema; pulmonary fibrosis; granuloma; and lymph follicle formation around the bronchioles using H&E specimens. The severity of each parameter (other than lymph follicle formation around the bronchioles) was assessed as follows: 0, no change; 1, weak; 2, moderate; 3, severe. Lymph follicle formation around the bronchioles was assessed as follows: 0, no change; 1, some infiltration; 2, with lymph follicle; 3, with granuloma. Each parameter was evaluated in the entire lung specimen; six sections of the lung lobes, including two sections of the left lobe and one section from each right lobe, were examined for all ratsunder 200× magnification. The scoring was performed by two authors (Y.N.-N. and M.Y.) in an unblinded manner. If the assessments differed, the two examiners evaluated the slide together and discussed the pathology until an agreement was reached.

To detect the quartz particles, polarised light microscopy (DP70 microscope with the Olympus Polarizer for Transmitted Light, U-P110; Olympus, Tokyo, Japan) was used. We observed entire specimens of the lungs under 200× magnification and evaluated the presence or absence of the quartz particles.

Other organs were examined histopathologically according to the INHAND criteria^45,46,50–53^ and the criteria published by Gary *et al*.^54^ using the H&E specimens. The histopathological analysis was performed by consultation with two authors (Y.N.-N. and K.I.).

### Statistics

Data are presented as means ± standard deviation of the mean (SD). The Dunnett’s test was used for comparisons of body and organ weights. The Tukey-Kramer test (multi-comparison test) was used to compare the numbers of the induced lung lesions and the inflammation scores. The Fisher’s exact probability test was applied to compare incidences of hyperplastic and neoplastic lesions of the lungs, liver, kidneys, spleen, skin, mammary glands, adrenal glands, and testes. Spearman’s rank correlation coefficient was used to compare the correlations between inflammation scores and the numbers of hyperplastic and neoplastic lesions per rat. P values less than 0.05 were considered to be significant. The JMP 8 software (SAS Institute Inc., Cary, NC, USA) was used for statistical processing.

## Supplementary information

Supplementary Information.

## Data Availability

All data generated or analysed during this study are included in this published article and its supplementary information files.
