# Supplementary material for: Single Intratracheal Quartz Instillation Induced Chronic Inflammation and Tumourigenesis in Rat Lungs
Source: Sci Rep. 2020 Apr 20;10:6647. doi: 10.1038/s41598-020-63667-4 (PMC7170867; doi:10.1038/s41598-020-63667-4)
Supplement: Supplementary file 1 — Supplementary Information. [file 41598_2020_63667_MOESM1_ESM.pdf]

Single Intratracheal Quartz Instillation Induced Chronic Inflammation and Tumorigenesis in Rat Lungs

Yuko Nakano-Narusawa[a], Masanao Yokohira[a], Keiko Yamakawa[a], Kousuke Saoo[a], b, Katsumi Imaida[a,*], Yoko Matsuda[a,*]

[a]Oncology Pathology, Department of Pathology and Host-Defence, Faculty of Medicine, Kagawa University, Kagawa 761-0793, Japan

[b]Kaisei General Hospital, Kagawa 762-0007, Japan

*To whom correspondence should be addressed

Yoko Matsuda, M.D., Ph.D.

E-mail: youkoh@med.kagawa-u.ac.jp

Oncology Pathology, Department of Pathology and Host-Defence, Faculty of Medicine, Kagawa University, 1750-1, Ikenobe, Miki-cho, Kita-gun, Kagawa, 761-0793 Japan

Phone, +81-87-891-2111; FAX, +81-87-891-2112

Katsumi Imaida, M.D., Ph.D.

E-mail: imaida@med.kagawa-u.ac.jp

Oncology Pathology, Department of Pathology and Host-Defence, Faculty of Medicine, Kagawa University, 1750-1, Ikenobe, Miki-cho, Kita-gun, Kagawa, 761-0793 Japan

Phone, +81-87-891-2111; FAX, +81-87-891-2112

Supplementary Data 1. Inflammation scores.

Groups	Neutrophil infiltration: In the alveolar wall	Neutrophil infiltration: In the alveolar space	Lymphocyte infiltration in the alveolar space	Macrophage infiltration in the alveolar space	Pulmonary oedema	Pulmonary fibrosis	Granuloma	Lymph follicle formation around bronchiole	Total score
52w-quartz	1.7 ± 0.7**	2.0 ± 0.5**	2.3 ± 0.7**	2.6 ± 0.5**,##	2.3 ± 0.7**	2.9 ± 0.3**,##	1.7 ± 0.7**	3.0 ± 0.0**	18.4 ± 2.5**,##
52w-saline	0.0 ± 0.0	0.2 ± 0.4	0.5 ± 0.5	1.0 ± 0.0	0.0 ± 0.0	0.3 ± 0.5	0.0 ± 0.0	1.2 ± 0.4	3.2 ± 1.2
96w-quartz	1.3 ± 0.6**	1.9 ± 0.9	2.4 ± 0.5**	1.2 ± 1.1	2.0 ± 0.9**	1.0 ± 0.8**	1.6 ± 0.7	3.0 ± 0.0**	14.4 ± 3.0 **
96w-saline	0.4 ± 0.9	1.0 ± 1.2	1.0 ± 0.0	0.8 ± 0.8	0.2 ± 0.4	0.0 ± 0.0	1.0 ± 0.0	1.8 ± 0.4	6.2 ± 2.8

**: Denote significant differences compared with the saline i.t. group (P<0.01)

##: Denote significant differences compared with Group 96w-quartz (P<0.01)

Supplementary Data 2. Incidences and numbers of hyperplastic and neoplastic lesions in the lungs.

Groups	Hyperplasia		Adenoma		Adenocarcinoma		Papilloma, Bronchiole	
	Incidence (%)	Number of lesions /rat	Incidence (%)	Number of lesions /rat	Incidence (%)	Number of lesions /rat	Incidence (%)	Number of lesions /rat
52w-quartz	0.0 (0/9)	0.0 ± 0.0	0.0 (0/9)	0.0 ± 0.0	0.0 (0/9)	0.0 ± 0.0	0.0 (0/9)	0.0 ± 0.0
52w-saline	0.0 (0/6)	0.0 ± 0.0	0.0 (0/6)	0.0 ± 0.0	0.0 (0/6)	0.0 ± 0.0	0.0 (0/6)	0.0 ± 0.0
96w-quartz	100 (21/21)	18.1 ± 6.6**	85.7 (18/21)**	2.1 ± 1.4**	81.0 (17/21)*	2.7 ± 2.1*	66.7 (14/21)	2.3 ± 2.7
96w-saline	100 (5/5)	5.6 ± 5.9	20.0 (1/5)	0.2 ± 0.4	20.0 (1/5)	0.2 ± 0.4	40.0 (2/5)	1.0 ± 1.4

*: Denote significant differences compared with the saline i.t. group (P<0.05)

**: Denote significant differences compared with the saline i.t. group (P<0.01)

Supplemental Data.3 Incidences of hyperplastic and neoplastic lesions of other organs.

Groups	52w-quartz	52w-saline	96w-quartz	96-saline
Liver				
Neoplastic lesions				
Cholangiocarcinoma	0.0 (0/9)	0.0 (0/6)	4.8 (1/21)	0.0 (0/5)
Non-neoplastic lesions				
Bile duct hyperplasia	100 (9/9)	100 (6/6)	100 (21/21)	100 (5/5)
Lymphocyte infiltration	100 (9/9)	100 (6/6)	85.7 (18/21)	100 (5/5)
Fibrosis	0.0 (0/9)	0.0 (0/6)	85.7 (18/21)	80.0 (4/5)
Fatty change	0.0 (0/9)	0.0 (0/6)	28.6 (6/21)	0.0 (0/5)
Basophilic focus	0.0 (0/9)	0.0 (0/6)	66.7 (14/21)	100 (5/5)
Clear cell focus	22.2 (2/9)	66.7 (4/6)	47.6 (10/21)**	80.0 (4/5)
Granulomatous	0.0 (0/9)	0.0 (0/6)	9.5 (2/21)	0.0 (0/5)
Cystic degeneration	0.0 (0/9)	0.0 (0/6)	0.0 (0/0)	20.0 (1/5)
Kidney				
Non-neoplastic lesions				
Tubule regeneration	55.6 (5/9)	33.3 (2/6)	85.7 (18/21)	80.0 (4/5)
Lymphocyte infiltration	33.3 (3/9)	50.0 (3/6)	14.3 (3/21)	0.0 (0/5)
Pyelonephritis	0.0 (0/9)	0.0 (0/6)	4.8 (1/21)	0.0 (0/5)
Casts	0.0 (0/9)	0.0 (0/6)	81.0 (17/21)	40.0 (2/5)
CPN	0.0 (0/9)	0.0 (0/6)	28.6 (9/21)*	80.0 (4/5)
Hemosiderin	0.0 (0/9)	0.0 (0/6)	90.5 (19/21)	100 (5/5)
Tubule necrosis	0.0 (0/9)	0.0 (0/6)	9.5 (2/21)	0.0 (0/5)
Hypertophy	0.0 (0/9)	0.0 (0/6)	4.8 (1/21)	0.0 (0/5)
Cyst	0.0 (0/9)	0.0 (0/6)	4.8 (1/21)	20.0 (1/5)
Congestion	0.0 (0/9)	0.0 (0/6)	4.8 (1/21)	0.0 (0/5)
Spleen				
Non-neoplastic lesions				
Necrosis	0.0 (0/9)	0.0 (0/6)	4.8 (1/21)	0.0 (0/5)
Congestion	0.0 (0/9)	0.0 (0/6)	66.7 (14/21)	100 (5/5)
Hemosiderin	0.0 (0/9)	0.0 (0/6)	61.9 (13/21)	100 (5/5)
Atrophy	0.0 (0/9)	0.0 (0/6)	0.0 (0/0)	20.0 (1/5)
Skin				
Neoplastic lesions				
Fibroma	0.0 (0/9)	0.0 (0/6)	14.3 (3/21)	0.0 (0/0)
Adenoma, sebaceous cell	0.0 (0/9)	0.0 (0/6)	4.8 (1/21)	0.0 (0/0)
Mammary gland				
Neoplastic lesions				
Adenocarcinoma	0.0 (0/9)	0.0 (0/6)	4.8 (1/21)	0.0 (0/0)
Adrenal gland				
Neoplastic lesions				
Pheochromocytoma, benign	0.0 (0/9)	0.0 (0/6)	23.8 (5/21)	0.0 (0/0)
Testis				
Neoplastic lesions				
Lydig cell tumour, adenoma	0.0 (0/9)	0.0 (0/6)	4.8 (1/21)	0.0 (0/0)
Non-neoplastic lesions				
Fibrosis	0.0 (0/9)	0.0 (0/6)	4.8 (1/21)	0.0 (0/0)
Atrophy	0.0 (0/9)	0.0 (0/6)	4.8 (1/21)	0.0 (0/0)

*: Denote significant differences compared with the saline i.t. group (P<0.05)

**: Denote significant differences compared with the saline i.t. group (P<0.01)

CPN: Chronic Progressive Nephropathy
